# The Doktabörse – an innovative online platform for research projects at the medical faculty of the LMU Munich

**DOI:** 10.3205/zma001107

**Published:** 2017-08-15

**Authors:** Leo Nicolai, Maximilian Gradel, Sofia Antón, Tanja Pander, Anke Kalb, Lisa Köhler, Martin R. Fischer, Konstantinos Dimitriadis, Philip von der Borch

**Affiliations:** 1 Klinikum der LMU München, Institut für Didaktik und Ausbildungsforschung in der Medizin, München, Germany; 2 Klinikum der LMU München, MeCuM-Mentor, München, Germany; 3 Klinikum der LMU München, MeCuM-Mentor, Doktamed, München, Germany; 4 LMU München, Neurologische Klinik und Poliklinik, München, Germany; 5 Klinikum der LMU München, Medzinische Klinik und Poliklinik IV, München, Germany

**Keywords:** Medical thesis, research project platform, doctoral thesis, research curriculum, online research platform, Doktabörse, DoktaMed

## Abstract

**Introduction: **One of the most important extracurricular aspects of medical studies in Germany is a research thesis completed by most students. This research project often times conveys relevant competencies for the physician’s role as scientist.

Nevertheless, the choice of the right project remains a challenge. Reasons for this are among others, missing structures for a comprehensive overview of research groups and their respective projects.

**Description of the project: **We developed the online platform *Doktabörse* as an online marketplace for doctoral research projects. The platform enables authorized researchers to create working groups and upload, deactivate and change research projects within their institute. For interested students, a front end with integrated search function displays these projects in a structured and well-arranged way. In parallel, the *Doktabörse* provides for a comprehensive overview of research at the medical faculty. We evaluated Researchers‘ and students‘ use of the platform.

**Results:** 96,6% of students participating in the evaluation (n=400) were in favor of a centralized research platform at the medical faculty. The platform grew at a steady pace and included 120 research groups in June 2016. The students appreciated the structure and design of the *Doktabörse.* Two thirds of all uploaded projects matched successfully with doctoral students via the platform and over 94% of researchers stated that they did not need technical assistance with uploading projects and handling the platform.

**Discussion**: The* Doktabörse* represents an innovative and well accepted platform for doctoral research projects. The platform is perceived positively by researchers and students alike. However, students criticized limited extent and timeliness of offered projects.

In addition, the platform serves as databank of research at the medical faculty of the LMU Munich. The future potential of this platform is to provide for an integrated management solution of doctoral thesis projects, possibly beyond the medical field and faculty.

## Zusammenfassung

**Einleitung: **Die Promotion ist ein wichtiger nicht curriculärer Bestandteil des Medizinstudiums und vermittelt oftmals relevante Kompetenzen für die Rolle des Arztes als Wissenschaftler. Trotzdem stellt die Wahl einer passenden Promotionsarbeit eine große Herausforderung dar.

Gründe hierfür sind unter Anderem fehlenden Strukturen zur übersichtlichen Darstellung der Arbeitsgruppen und der Promotionsprojekte.

**Projektbeschreibung: **Wir konzipierten die Online-Promotionsplattform *Doktabörse* als Instrument zur Vermittlung von Doktorarbeiten. Diese ermöglicht es zugangsberechtigten Forschenden innerhalb einer Institutsstruktur Arbeitsgruppen anzulegen und Promotionsarbeiten auszuschreiben, anzupassen und zu deaktivieren. In einem Frontend mit integrierter Suchfunktion werden diese Angebote dann strukturiert und übersichtlich für interessierte Studierende dargestellt. Gleichzeitig bietet die *Doktabörse* einen Überblick über die Forschungslandschaft der Medizinischen Fakultät. Die Nutzung durch Forschende und Studierende wurde evaluiert.

**Ergebnisse: **96,6% der an einer Umfrage (n=400) teilnehmenden Studierenden sprachen sich für eine zentrale Promotionsplattform an der Fakultät aus. Die Plattform verzeichnet ein stetes Wachstum und umfasst 120 Arbeitsgruppen (Stand Juni 2016). Die Studierenden lobten die Struktur, die visuelle Gestaltung und die Übersichtlichkeit der *Doktabörse*. Zwei Drittel der Inserate führten zu einer erfolgreichen Vermittlung von Doktoranden und über 94% der teilnehmenden Forschenden gaben an, keine technische Hilfe bei der Ausschreibung von Promotionen zu benötigen.

**Diskussion: **Die *Doktabörse* stellt ein innovatives und gut akzeptiertes Instrument zur Vermittlung von Doktorarbeiten dar. Sie wird von Forschenden wie auch Studierenden positiv aufgenommen, allerdings bemängeln Letztere den zu geringen Angebotsumfang und die Aktualität. Zusätzlich dient sie als Datenbank der forschenden Institute an der Medizinischen Fakultät der LMU München. Das Potential dieser Plattform ist es, fach- und fakultätsübergreifend eine integrative Lösung für das Management von Promotionsvorhaben zu bieten.

## Introduction

For the majority of medical students, a concomitant medical thesis project is the gateway to medical research and therefore also conveys relevant competencies for the physician’s role as scientist [1]. Problems are the inhomogeneous quality of research projects as well as high drop-out quotas [2], [3], [4], [5]. Previous studies have shown that mainly inadequate information, insufficient assistance by as well as non-availability of the supervisor, conceptual deficits, slow or absent progress and inadequate time and effort is responsible for this [6], [7], [8]. 

In this regard, medical students at the LMU criticized among other things that there was no systematic and comprehensive overview of doctoral research projects at the faculty [9], as the inflexible hospital’s website is infrequently used for announcing open positions or projects. 

Many other German medical faculties do not have any (i.e. Greifswald, Dresden) or only static websites as the LMU has (i.e. Frankfurt, Hamburg) to present research projects to interested medical students. 

Only two platforms enable researchers to actively manage their own research project offers (Jena and Münster). However, these two platforms don’t include an institute database listing working groups and core research fields. 

We formulated the hypothesis that a structured online presentation of offered doctoral research projects, institutes and working groups could contribute to a more informed and therefore better choice of an appropriate project. Therefore, the goal of our project was to develop an online platform for doctoral research projects. 

## Project description

The *Doktabörse* was created in May 2014 to serve as a permanent platform for the search doctoral research projects at the medical faculty of the LMU Munich. The goals of the initial version of the *Doktabörse* are:

1. Provide for a comprehensive databank of all institutes and working groups at the medical faculty of the LMU Munich active in research.
2. To facilitate interaction of researchers and interested students.
3. To provide for a comprehensive offer of doctoral research projects for medical students.
4. To present these projects in a standardized, up-to-date and clear way.

On the one hand, the platform is intended to provide a useful, simple and straightforward website for both students and researchers, while at the same time limiting the administrative burden for website administrators.

### Databank und structure

All institutes that contribute to research at the medical faculty of the LMU Munich were added to the databank of our platform as “institutes”, including a short description.

**Figure 1:**
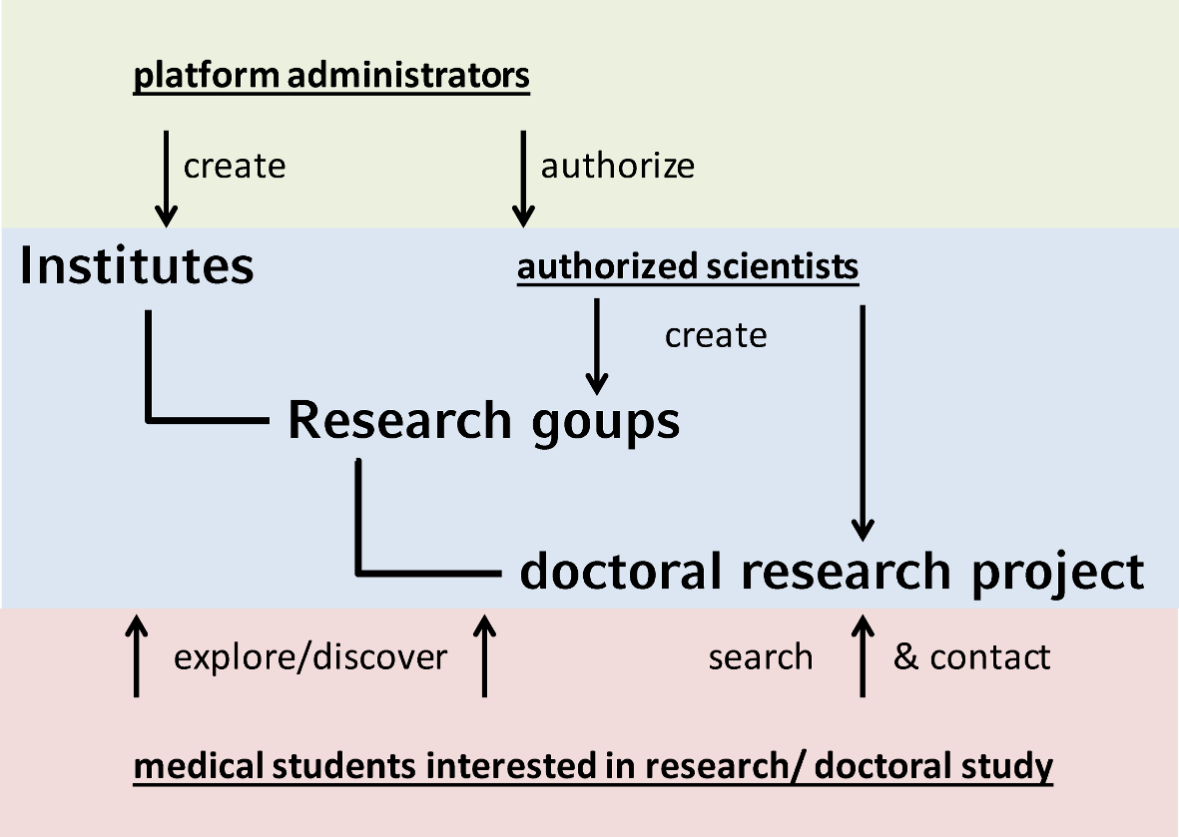
Structure and function of the databank Doktabörse. The hierarchy consists of institutes->research groups-> doctoral research studies. The website’s administrators create institutes and authorize scientists. Authorized scientists in turn create groups and offer doctoral research studies. Students can browse through institutes and groups and/or search and contact projects directly.

Our next step was to contact all institute directors and group leader known to us, and provide them with individual access codes to the institute management backend (Hierarchical structure of the *Doktabörse*: see Figure 1 (Fig. 1)). 

After logging in, institute directors and group leaders were able to create their individual working group, including a description, within their respective institute environment. In a second step, they were then able to upload offers for doctoral research projects. 

As a result, researchers could create working groups and upload research projects in minutes, which in turn increased usability of the *Doktabörse* for institutes and limited the administrative burden. The responsible person can modify, deactivate or delete the uploaded research projects as well as the working group details at any time. 

**Figure 2:**
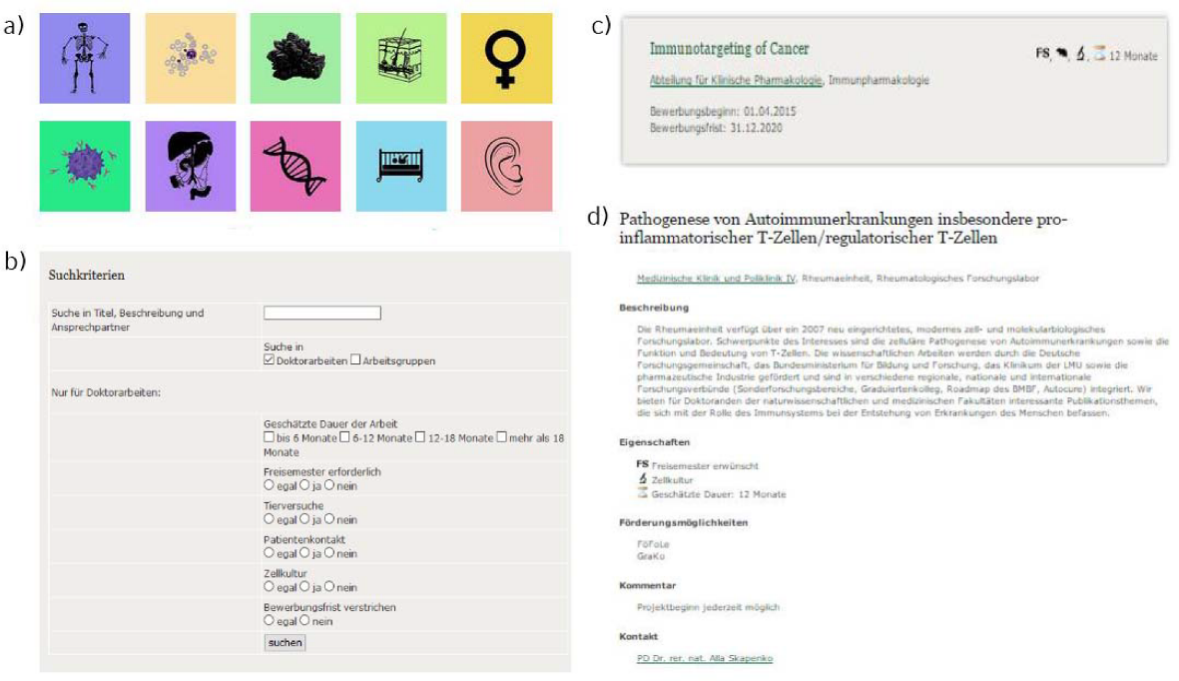
1. Tile-based front end of the institutes‘ and research groups‘ databank. The tiles are theme-based and link to a list of research groups working in the respective field. 2. Search function for uploaded research projects/groups. The searchable criteria include key words in research groups and projects, as well as estimated duration of the project, involvement of patients or animal experiments, cell culture and the necessity of full time research. 3. Short description “ad” of an exemplified doctoral research project. The properties of the project are displayed by icons in addition to the written out title, respective institute and application deadline. 4. Long description of an exemplified doctoral research project. This view includes a description of the topic, a comment box the respective researcher, and his or her contact details.

Every offered research project includes a description, as well as properties and type of research (see Figure 2 (Fig. 2), Point d).

#### User interface

An attractive and timely frontend interface contributes to the success of a website in a major fashion. We therefore created two separate but closely linked interfaces: One to view all uploaded, active research project offers and a second interface to view or browse institutes and working groups. 

The research project interface offers a list of the uploaded, active projects in a short “overview” format. In addition, a search field and intuitive symbols, which visually present the key facts about each project, add extra user friendliness. If a project is of potential interest, clicking on the offer will reveal an extended description and contact details for getting into touch with the respective researcher (see Figure 2 (Fig. 2), Point b).

The institute and research group interface includes a drop-down menu, through which any of the institutes that are part of the databank can be selected. Every individual institute site includes a description, the respective director, and a detailed listing of working groups as well as research projects. Alternatively, one can utilize an intuitive tile based interface which displays core research fields from cancer to neuroscience in a representative way, to gain an overview over research performed at the medical faculty of the LMU Munich (see Figure 2 (Fig. 2), Point a). 

#### Integration

In addition to serve as a platform for potential research projects, the *Doktabörse* has the goal to support students with relevant information on research and doctoral theses. Therefore, we included links to the official website of the faculty as well as a link to a researcher-student agreement form. In addition, the platform is integrated into the *DoktaMed* website, and offers direct access to important information.

## Results

In spring 2016, 486 researchers working in 47 institutes had been authorized on the *Doktabörse*. The webpage had 17.400 visitors from January 2016 until June 2016, which averages to more than 100 users a day. This represents a positive trend compared to the previous year. 

To evaluate the development of this new platform, we collected key statistics of the platform at three time points over the course of one year. At every time point, we found a stable number of active and up-to-date doctoral research projects on offer (17±1). After the start-up phase of the *Doktabörse *(5 months) 60% of institutes had created active research groups. This index of activity was boosted by 16.6%, increasing to 70% in January 2016. The total number of working groups rose by 33% from 90 (12/2014) to 120 (1/2016) over the same time period.

96,6% of students participating in the evaluation (n=400) were explicitly in favor of a centralized research platform at the medical faculty. Students‘ free text comments lauded *Doktabörse* for its structure and design („easy to grasp structure, easy to handle“). The *Doktabörse* received an overall grade of 2.7(±1.1, 1= excellent, 6=failure). Students were critical about up-to-datedness, rated with Grade 3.6(±1.5) und diversity of offered projects, Grade 4.1(±1.3). They positively evaluated user friendliness with grade 2.3(±1.1) and the clear arrangement of information with grade 2.5(±1.1). Numerous free text comments praised “the platform per se and the underlying concept”. However, they wished for “more and up-to-date offers as well as more non-experimental research projects”. 

The results of the evaluation targeting institute and research group leaders showed that 80,1% of them were already familiar with the platform *Doktabörse*. A subset of 52% used the platform to upload doctoral research projects. In 83% of cases, at least one student contacted the respective researcher concerning his project, and in 80% of cases this then led to an agreement, with a student signed on the project. This amounts to successful matching in two thirds of cases. Researchers The overall grade for the platform by researchers was 1,73(±0.75) on a Likert scale from 1=excellent to 6=failed. Free text comments appreciated “the clear and logical presentation of topics and projects”, the “centralized collection of all research projects” and the “relatively simple management of groups and projects”.

## Discussion

Our goal was to implement an innovative and modern platform for doctoral research projects at the medical faculty of the LMU Munich. Students as well as researchers strongly support the development of such a platform. Our data provides some evidence for successful matching of researchers and students via the *Doktabörse*. In addition, our platform allows for good planning and display of active interest in students, both important factors for the success of medical theses [9]. The Web 2.0 makes it possible that researchers can upload projects at any time, as well as self-administration of groups and projects. 

This in turn guarantees direct and quick interaction of researchers and students, lifting administrative burden off the faculty or hospital [10]. All user groups evaluate the Doktabörse as intuitive and straightforward, and it seems to influence the choice of research projects in a positive fashion. Somewhat in contrast to the good evaluation and content of all users stands the just average evaluation of the *Doktabörse* by students. Qualitative analysis of free text comments hints at the fact that this is originating from limited range of projects offered as well as a low percentage of clinical research projects. 

We view the *Doktabörse* as an important platform for the presentation of potential doctoral research projects at the medical faculty of the LMU Munich. The *Doktabörse* is implemented in Open Source Software, which enables for a direct integration of this platform at other faculties, as well as easy adaptation to the individual needs of other faculties in this process. 

## Competing interests

The authors declare that they have no competing interests.
